# Positive cooperativity in synergistic activation of Wnt proteins

**DOI:** 10.1007/s11033-024-09831-9

**Published:** 2024-08-18

**Authors:** Clemence Bonnet, Christiana S. Han, Sophie X. Deng, Jie J. Zheng

**Affiliations:** 1 Stein Eye Institute, University of California, Los Angeles, CA 90095 USA; 2 INSERM, UMRS1138, Team 17, From Physiopathology of Ocular Diseases to Clinical Development, Université Paris Cité, Centre de Recherche des Cordeliers, Paris, France; 3 Molecular Biology Institute, University of California, Los Angeles, CA USA

**Keywords:** Cooperative binding, Signalosome, Wnt3a, Wnt mimic, Wnt/b-catenin signaling

## Abstract

**Background:**

Wnt proteins are crucial for embryonic development, stem cell growth, and tissue regeneration. Wnt signaling pathway is activated when Wnt proteins bind to cell membrane receptors.

**Methods and results:**

We employed a luciferase reporter assay in HEK293STF cells to measure Wnt protein-induced signaling. We observed that Wnt3a uniquely promotes the Wnt/β-catenin pathway through positive cooperativity. Additionally, MFH-ND, a molecular mimic of Wnt ligands, markedly increased Wnt3a-induced signaling in a dose-responsive manner. This suggests that various Wnt ligands can synergistically enhance Wnt pathway activation.

**Conclusions:**

The study suggests the likelihood of various Wnt ligands coexisting in a single signalosome on the cell membrane, providing new insights into the complexities of Wnt signaling mechanisms.

## Introduction

Wnt/β-catenin signaling pathways play a crucial role in embryonic development, as well as in stem cell proliferation, differentiation, self-renewal, tissue regeneration, and remodeling in adults [1, 2]. In the absence of Wnt signaling, phosphorylated β-catenin undergoes continuous proteasomal degradation. This degradation is facilitated by a destruction complex consisting of adenomatous polyposis coli, Axin, casein kinase 1α, and glycogen synthase kinase 3β [3]. Activation of Wnt/β-catenin signaling occurs when Wnt proteins bind to both Frizzled (FZD) and low-density lipoprotein receptor-related proteins 5 or 6 (LRP5/6) transmembrane receptors simultaneously [4]. This activation leads to an increase in intracellular β-catenin levels, resulting in alterations in gene expression and cellular behavior [5].

Wnt proteins, being lipid-modified, possess hydrophobic properties and are relatively insoluble [6]. Consequently, their range of action is limited, but they exhibit potent morphogenic effects. Notably, the concentration gradient of Wnt proteins within the stem cell niche plays a crucial role in embryonic development and stem cell renewal in vivo [7]. However, the detailed correlation between extracellular Wnt protein concentration and intracellular Wnt signaling activity remains unexplored. Furthermore, similar to other cellular signaling pathways, binding of Wnt proteins to their receptors, FZDs and LRP5/6 can induce the formation of signalosomes—large transmembrane oligomer complexes [8]. Yet, the impact of Wnt signalosome formation on the strength of Wnt signaling activity within cells remains unclear. Additionally, there are nineteen Wnt proteins in humans and other mammals [5, 6], and they are often expressed simultaneously [9, 10]. The synergistic effects of different Wnt proteins in organ development [10] and intracellular signaling [11] have been reported [12–14]. Nevertheless, the interplay between different Wnt proteins and its influence on Wnt signalosome formation and intracellular Wnt activity remain unclear.

## Results

In the current study, we quantified the Wnt signaling activity induced by Wnt proteins using a luciferase reporter-based cellular assay in HEK293STF cells. These cells are a stably transfected HEK293 cell line expressing a luciferase reporter controlled by seven LEF/TCF binding sites (Super TOP-Flash) [15].

**Fig. 1:**
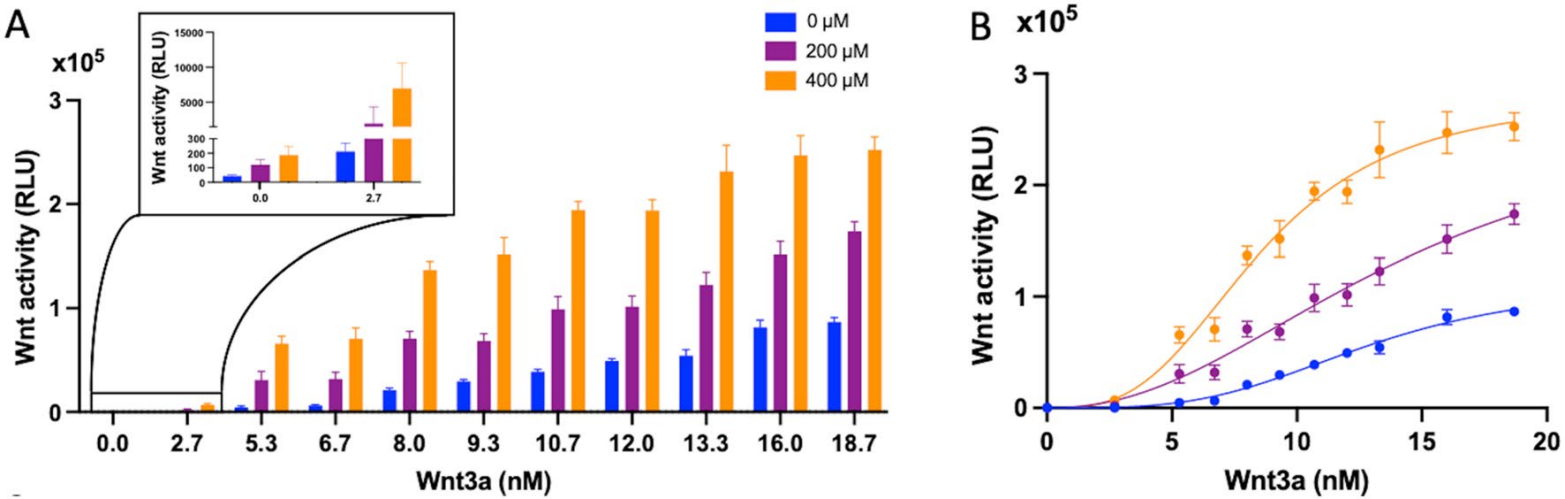
Wnt activation in HEK293STF cells measured by TOP-Flash assay. **A** Luciferase-based TOP-Flash assay demonstrating the binding capacity of Wnt3a, MFH-ND, and the combination of Wnt3a + MFH-ND at increasing concentrations. Results show the mean ± SEM of three independent experiments performed in triplicates and are presented as relative light units (RLU) of fluorescence intensity to firefly luciferase. **B** Non-linear regression model fitting of the Wnt activation curve in the presence of MFH-ND, using the formula: Y = X^h^ × X_Max_/(X^h^ + EC_50_^h^). The curve fitting analysis was conducted with GraphPad Prism 9 v9.3.1, with h, X_Max_, and EC_50_ representing the three curve fitting parameters

**Fig. 2:**
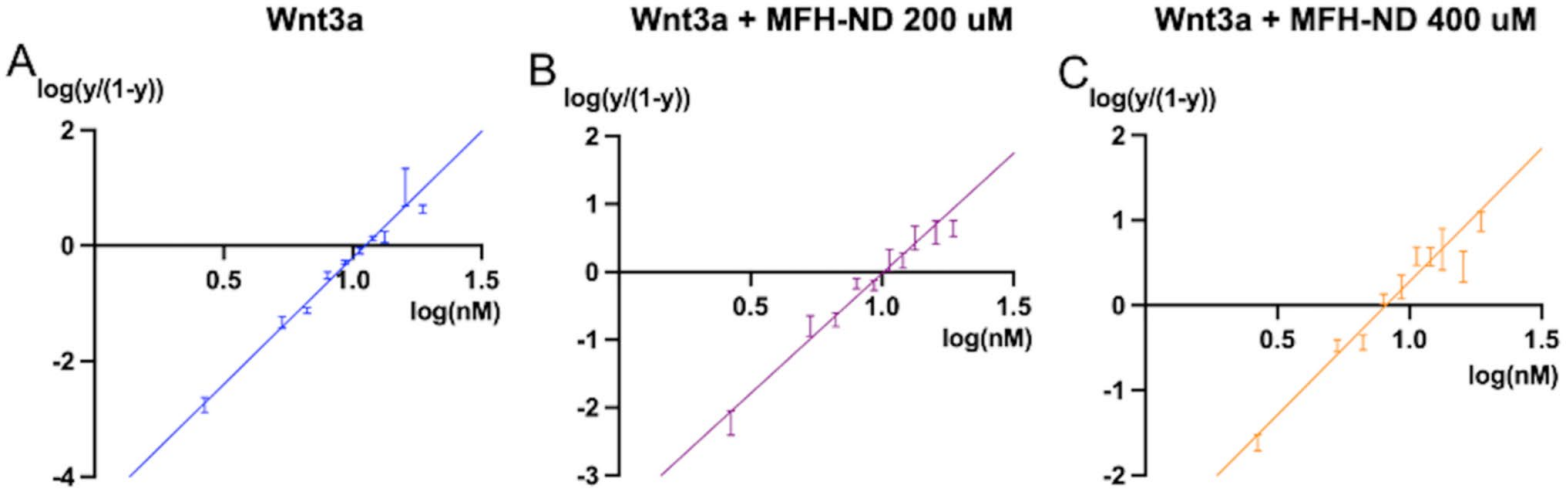
Hill factors determined by Hill plot analysis. Hill plots were generated to calculate Hill factors for **A** Wnt3a alone, **B** Wnt3a with 200 mM MFH-ND, and **C** Wnt3a with 400 mM MFH-ND. The Hill–Langmuir equation, log[y(1 − y)] versus log [x] [18], was employed to construct the hill plot and obtain the hill coefficient. In the equation, y represents Wnt activation measured in the TOP-Flash assay, while x corresponds to the concentration of Wnt3a. The slope of the plotted line represents the Hill coefficient, or Hill factor, and the standard error indicates the error in the coefficient estimation

To induce Wnt activity, we treated the HEK293STF cells with recombinant Wnt3a. Upon analyzing the Wnt activity within the cells by measuring luciferase activity, we observed that the Wnt activation induced by Wnt3a was not a single-phase response (Figs. 1A, B). Curve fitting analysis revealed a calculated Hill factor of 3.5 ± 0.5 (Fig. 2). A Hill factor greater than 1.0 signifies positive cooperation [16], our data indicates strong positive cooperation in Wnt activation by Wnt3a within the cells.

To investigate if the synergistic activity of different Wnt proteins exhibits positive cooperation, we employed a Wnt mimic small-molecule called MFH-ND, as there was a lack of reliable recombinant canonical Wnt proteins apart from Wnt3a. MFH-ND was designed by combining a small molecule, MFH, which binds to the cysteine-rich domain (CRD) of FZD, with a peptide derived from the N-terminal of Dickkopf-1 (DKK1) protein, named ND, which binds to the first β-propeller domain of LRP5/6. This construct enables MFH-ND [17] to mimic Wnt proteins in a broad spectrum manner.

Although MFH-ND exhibits broad binding capabilities to different FZD-CRDs and the first β-propeller domains of both LRP5 and LRP6, it is not as potent as Wnt3a in activating Wnt/β-catenin signaling in HEK293STF cells. Our experiments showed that the Wnt activity induced by 400 μM MFH-ND in the cells is comparable to the activity induced by 2.7 nM Wnt3a. Therefore, to mimic a Wnt protein, we utilized two concentrations of MFH-ND: 200 μM and 400 μM, respectively.

We observed that MFH-ND alone demonstrated limited ability to activate Wnt signaling in the cells at the tested concentration. However, when combined with Wnt3a, MFH-ND significantly enhanced Wnt3a-induced Wnt activity, suggesting a synergistic effect between the two. Remarkably, the combined effect of Wnt3a and MFH-ND surpassed the sum of their individual effects at equivalent concentrations. Curve fitting analysis revealed that Wnt3a alone induced a maximum Wnt activity of 1.1 ± 0.1 × 10^5^. In contrast, the presence of 200 μM and 400 μM MFH-ND with Wnt3a resulted in maximum Wnt activities of 2.7 ± 0.8 × 10^5^ and 2.8 ± 0.2 × 10^5^, respectively (Fig. 1B). Furthermore, our observations exhibited Hill factors exceeding 1.0 when fitting the curves of Wnt3a-induced Wnt activity at both MFH-ND concentrations. The calculated Hill factor was 2.2 ± 0.5 in the presence of 200 μM MFH-ND and 3.0 ± 0.5 in the presence of 400 μM MFH-ND.

To further confirm the accuracy of the calculated Hill factors obtained through curve fitting, we conducted a Hill plot analysis [18] for all three conditions: Wnt3a alone, Wnt3a with 200 μM MFH-ND, and Wnt3a with 400 μM MFH-ND (Fig. 2). Our analysis revealed Hill factors of 3.5 ± 0.5, 2.2 ± 0.5, and 3.0 ± 0.5, respectively, which aligns with the results obtained from curve fitting. These findings provide additional evidence that the synergistic effect of Wnt3a and MFH-ND on Wnt signaling induction is a positively cooperative event.

## Discussion

**Fig. 3:**
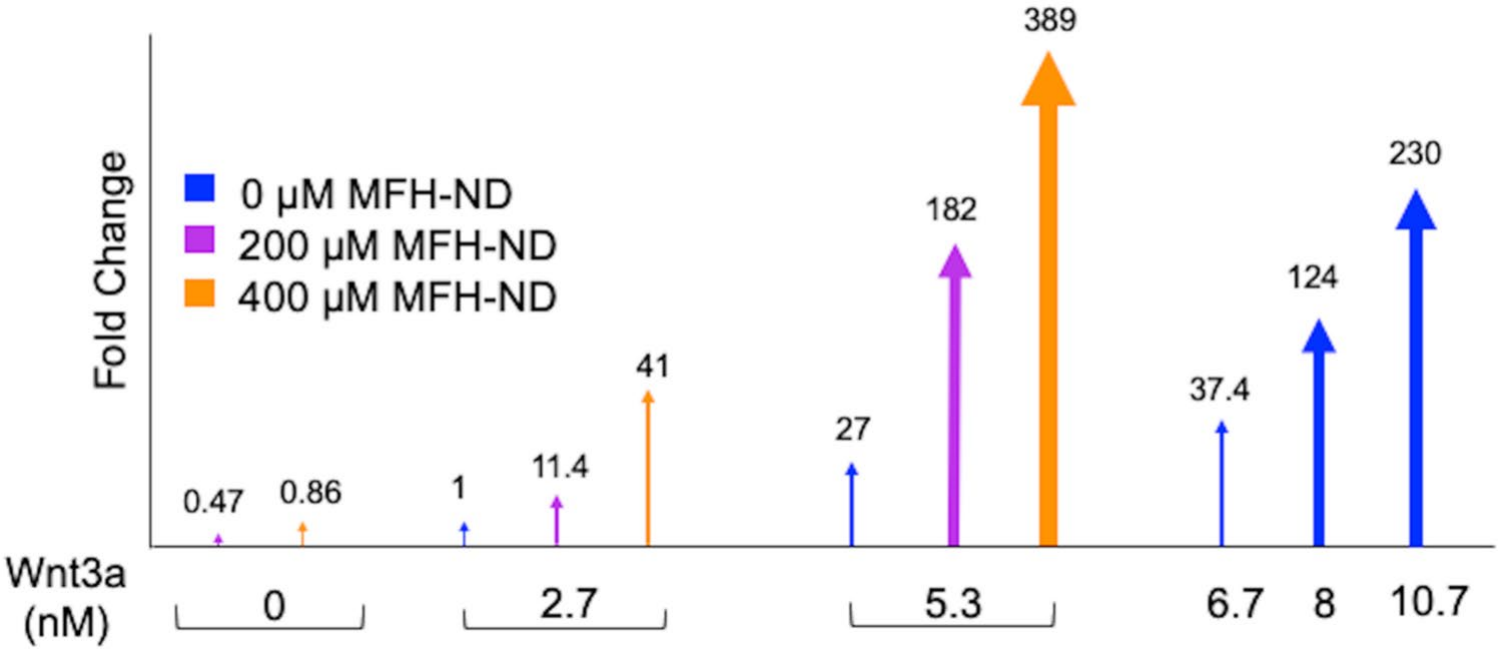
Binding capacity of MFH-ND expressed as fold change (Wnt3a at 2.7 nM). Graphical representation of luciferase assay showing the binding capacity of Wnt3a, MFH-ND, and the combination of Wnt3A + MFH-ND at increasing concentrations

According to the concept that Wnt proteins interact with Wnt receptors, leading to the formation of Wnt signalosomes at the cell membrane [19, 20], our findings support the notion that the initiation of Wnt3a-induced Wnt signalosomes follows a positively cooperative mechanism. Particularly, the effect of Wnt signalosomes is most pronounced during the early stages of their formation. In our study, we observed a 27-fold increase in Wnt activity inside the cells when the concentration of Wnt3a was doubled, indicating that a minimum concentration of 5.3 nM Wnt3a is sufficient to induce Wnt signalosome formation (Fig. 3). However, due to the positive cooperativity of the process, we further observed an additional 8.5-fold enhancement in cellular Wnt activity when the Wnt3a concentration was doubled from 5.3 to 10.7 nM. To address the possible synergistic interactions between different Wnt proteins [10, 11], we utilized the molecule MFH-ND as a mimic of another Wnt ligand [17]. While MFH-ND alone demonstrated low potency in activating Wnt signaling in the Top-Flash assay, we observed that at a concentration of 400 µM, it induced similar Wnt activity as Wnt3a at 2.7 nM. This implies that 400 µM MFH-ND possesses equivalent potency to another Wnt protein at 2.7 nM. Remarkably, when combining 2.7 nM Wnt3a with 200 µM MFH-ND, we observed significantly higher Wnt activity (41-fold) compared to Wnt3a alone at 5.3 nM (27-fold). Furthermore, the augmented Wnt activity induced by the MFH-ND and Wnt3a combination, relative to the equivalent concentration of Wnt3a alone, remained consistent across different concentrations. For instance, the combination of 5.3 nM Wnt3a and 400 µM MFH-ND resulted in a 389-fold increase in Wnt activation, whereas 8 nM of Wnt3a alone induced only 124-fold Wnt activity (Fig. 3).

**Fig. 4:**
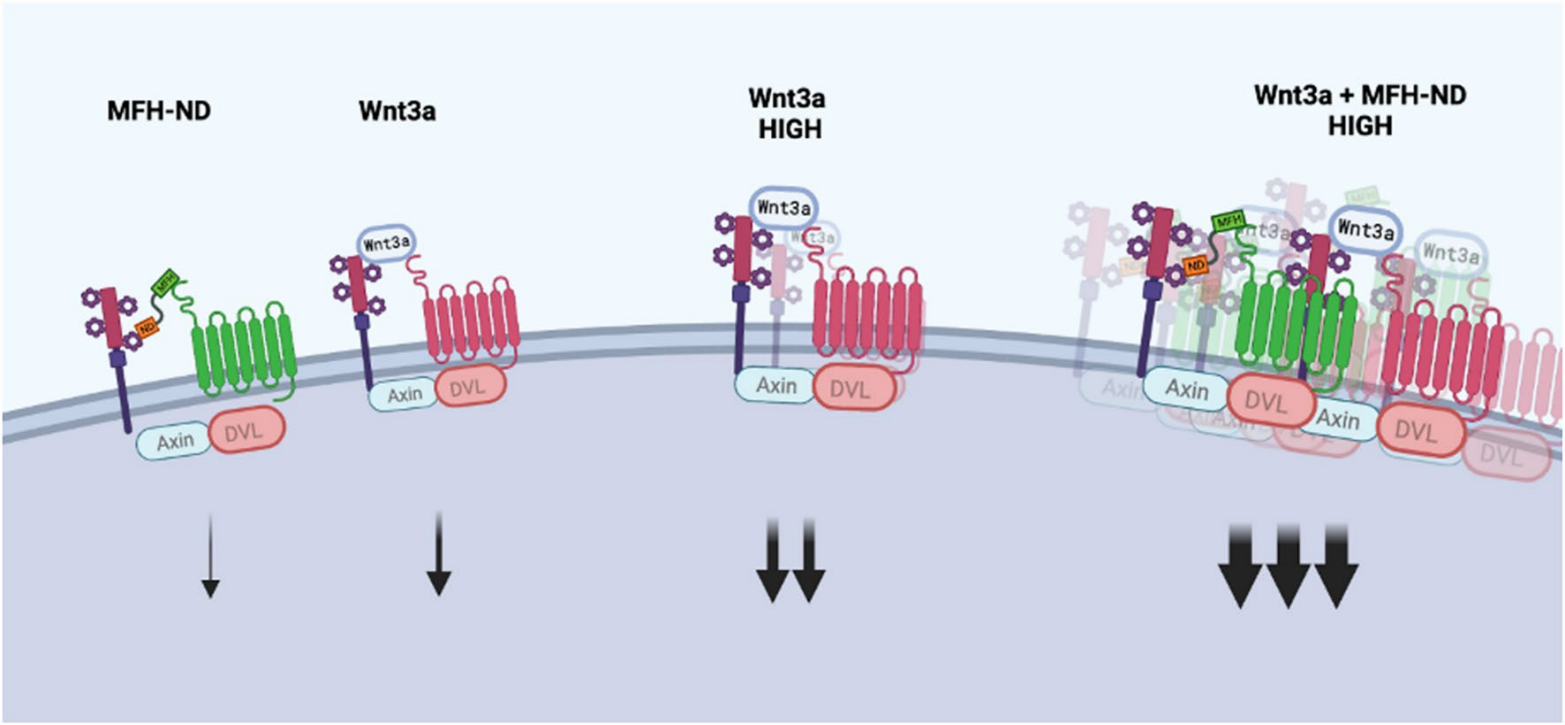
Working model illustrating the dynamic process of signalosome assembly triggered by multiple Wnt proteins. The model shows the synergistic effect of multiple canonical Wnt proteins, leading to the formation of larger signalosomes on the cell membrane. This results in enhanced intracellular Wnt signaling, potentially attributed to the binding of different Wnt proteins to various FZD receptors

Based on the concept of Wnt signalosome [8], the study suggests that multiple canonical Wnt proteins can synergistically work together to form larger signalosomes on the cell membrane, resulting in stronger intracellular Wnt signaling (Fig. 4). This phenomenon is likely due to the binding of multiple different Wnt proteins to different FZD receptors. Consequently, the presence of different Wnt proteins within a Wnt signalosome brings additional FZD receptors to the signalosome, creating a larger signalosome (Fig. 4).

Therefore, it is possible that the concentration gradient of canonical Wnt proteins plays a crucial role in controlling the size and stability of the Wnt signalosome. Higher concentrations of canonical Wnt proteins promote the formation of larger signalosomes and increase their stability. Thus, higher concentrations not only induce higher Wnt activity but also prolong the duration of the induced Wnt activity.

In summary, our study illuminates the robust morphogenic properties associated with canonical Wnt proteins [1], given their pivotal role in regulating stem cell renewal, tissue homeostasis, and tissue repair [1]. These effects are underpinned by the positive cooperation observed during Wnt signalosome formation. In the context of cancer, the formation of an irregular Wnt signalosome can contribute to the stabilization of aberrant signaling due to formation of large Wnt signalosomes. Therefore, our finding in the current study underscores the challenges involved in developing therapeutic inhibitors that specifically target abnormal Wnt signaling pathways in cancer [21].

## Materials and methods

To carry out cell-based Wnt luciferase reporter assay, stably transfected HEK293 luciferase-based reporter cell line, HEK293STF (CRL-3249, ATCC, Manassas, VA), which expresses firefly luciferase under the control of T-cell factor/lymphoid enhancer factor (TCF/LEF) promoter [15], were used to examine the activation of the small molecule MFH-ND on activating the canonical Wnt signaling pathway. HEK293STF cells were cultured in 5% CO_2_ at 37 °C in Dulbecco’s modified Eagle’s medium supplemented with 4.5 g/L d-glucose and 2 mM glutamine (DMEM, Invitrogen, Carlsbad, CA) containing 10% fetal bovine serum (FBS, Invitrogen), 0.1 mM nonessential amino acids (Gibco), and 10 mM HEPES (Gibco). The cells were seeded at 2 × 10^5^ cells/well in a 96-well plate (Corning) and incubated overnight, and then were treated with vehicle (0.1% dimethyl sulfoxide (DMSO); Sigma-Aldrich), 0–18.7 nM recombinant human Wnt3a protein with carrier (5036-WN-010, R&D, Minneapolis, MN), 0–400 µM of MFH-ND, or Wnt3a (0–18.7 nM) with MFH-ND (0–400 µM), for 18 h. Cell viability and firefly luciferase activity were measured using the ONE-Glo™ + Tox Luciferase Reporter and Cell Viability Assay kit (#E7120, Promega, Madison, WI). Microplate Reader, FilterMax F5 (Molecular Devices, Sunnyvale, California) was used to measure cell viability and firefly luciferase activity. The Wnt pathway activity was expressed as the relative light unit (RLU) of fluorescence intensity to firefly luciferase. Three individual experiments were performed in triplicate.

## Data Availability

No datasets were generated or analysed during the current study.

## References

[1] Nusse R, Clevers H (2017) Wnt/beta-catenin signaling, disease, and emerging therapeutic modalities. Cell 169:985–999. 10.1016/j.cell.2017.05.016 28575679 10.1016/j.cell.2017.05.016

[2] Bonnet C, Brahmbhatt A, Deng SX, Zheng JJ (2021) Wnt signaling activation: targets and therapeutic opportunities for stem cell therapy and regenerative medicine. RSC Chem Biol 2:1144–1157. 10.1039/d1cb00063b 34458828 10.1039/d1cb00063b PMC8341040

[3] Law SM, Zheng JJ (2022) Premise and peril of Wnt signaling activation through GSK-3beta inhibition. iScience 25:104159. 10.1016/j.isci.2022.104159 35434563 10.1016/j.isci.2022.104159 PMC9010644

[4] van Amerongen R (2012) Alternative Wnt pathways and receptors. Cold Spring Harb Perspect Biol. 10.1101/cshperspect.a007914 22935904 10.1101/cshperspect.a007914 PMC3475174

[5] Cadigan KM, Nusse R (1997) Wnt signaling: a common theme in animal development. Genes Dev 11:3286–3305 9407023 10.1101/gad.11.24.3286

[6] Willert K, Nusse R (2012) Wnt proteins. Cold Spring Harb Perspect Biol 4:a007864. 10.1101/cshperspect.a007864 22952392 10.1101/cshperspect.a007864 PMC3428774

[7] Angers S, Moon RT (2009) Proximal events in Wnt signal transduction. Nat Rev Mol Cell Biol 10:468–477. 10.1038/nrm2717 19536106 10.1038/nrm2717

[8] Bilic J, Huang YL, Davidson G et al (2007) Wnt induces LRP6 signalosomes and promotes dishevelled-dependent LRP6 phosphorylation. Science 316:1619–1622 17569865 10.1126/science.1137065

[9] Nakatsu MN, Ding Z, Ng MY, Truong TT, Yu F, Deng SX (2011) Wnt/beta-catenin signaling regulates proliferation of human cornea epithelial stem/progenitor cells. Invest Ophthalmol Vis Sci 52:4734–4741. 10.1167/iovs.10-6486 21357396 10.1167/iovs.10-6486 PMC3175950

[10] Alok A, Lei Z, Jagannathan NS et al (2017) Wnt proteins synergize to activate beta-catenin signaling. J Cell Sci 130:1532–1544. 10.1242/jcs.198093 28289266 10.1242/jcs.198093

[11] Miller MF, Cohen ED, Baggs JE, Lu MM, Hogenesch JB, Morrisey EE (2012) Wnt ligands signal in a cooperative manner to promote foregut organogenesis. Proc Natl Acad Sci USA 109:15348–15353. 10.1073/pnas.1201583109 22949635 10.1073/pnas.1201583109 PMC3458331

[12] Colozza G, Koo BK (2021) Wnt/beta-catenin signaling: structure, assembly and endocytosis of the signalosome. Dev Growth Differ 63:199–218. 10.1111/dgd.12718 33619734 10.1111/dgd.12718 PMC8251975

[13] Kang K, Shi Q, Wang X, Chen YG (2022) Dishevelled phase separation promotes Wnt signalosome assembly and destruction complex disassembly. J Cell Biol. 10.1083/jcb.202205069 36342472 10.1083/jcb.202205069 PMC9811998

[14] Cristobal CD, Ye Q, Jo J et al (2021) Daam2 couples translocation and clustering of Wnt receptor signalosomes through Rac1. J Cell Sci. 10.1242/jcs.251140 33310913 10.1242/jcs.251140 PMC7860116

[15] Xu Q, Wang Y, Dabdoub A et al (2004) Vascular development in the retina and inner ear: control by Norrin and Frizzled-4, a high-affinity ligand-receptor pair. Cell 116:883–895 15035989 10.1016/s0092-8674(04)00216-8

[16] Stefan MI, Le Novere N (2013) Cooperative binding. PLoS Comput Biol 9:e1003106. 10.1371/journal.pcbi.1003106 23843752 10.1371/journal.pcbi.1003106 PMC3699289

[17] Zhang C, Mei H, Robertson SYT, Lee HJ, Deng SX, Zheng JJ (2020) A small-molecule Wnt Mimic improves human limbal stem cell ex vivo expansion. iScience 23:101075. 10.1016/j.isci.2020.101075 32361505 10.1016/j.isci.2020.101075 PMC7200314

[18] Gesztelyi R, Zsuga J, Kemeny-Beke A, Varga B, Juhasz B, Tosaki A (2012) The Hill equation and the origin of quantitative pharmacology. Arch Hist Exact Sci 66:427–438. 10.1007/s00407-012-0098-5

[19] Case LB, Ditlev JA, Rosen MK (2019) Regulation of transmembrane signaling by phase separation. Annu Rev Biophys 48:465–494. 10.1146/annurev-biophys-052118-115534 30951647 10.1146/annurev-biophys-052118-115534 PMC6771929

[20] Schubert A, Voloshanenko O, Ragaller F et al (2022) Superresolution microscopy localizes endogenous Dvl2 to Wnt signaling-responsive biomolecular condensates. Proc Natl Acad Sci USA 119:e2122476119. 10.1073/pnas.2122476119 35867833 10.1073/pnas.2122476119 PMC9335300

[21] Tran FH, Zheng JJ (2017) Modulating the Wnt signaling pathway with small molecules. Protein Sci. 10.1002/pro.3122 28120389 10.1002/pro.3122 PMC5368067

